# Antitumor Activity of Pulvomycin via Targeting Activated-STAT3 Signaling in Docetaxel-Resistant Triple-Negative Breast Cancer Cells

**DOI:** 10.3390/biomedicines9040436

**Published:** 2021-04-17

**Authors:** Woong Sub Byun, Eun Seo Bae, Jinsheng Cui, Hyen Joo Park, Dong-Chan Oh, Sang Kook Lee

**Affiliations:** College of Pharmacy, Natural Products Research Institute, Seoul National University, Seoul 08826, Korea; sky_magic@naver.com (W.S.B.); ddol1289@snu.ac.kr (E.S.B.); cuijs@snu.ac.kr (J.C.); phj00@snu.ac.kr (H.J.P.); dongchanoh@snu.ac.kr (D.-C.O.)

**Keywords:** signal transducer and activator of transcription 3, triple-negative breast cancer, resistance, metastasis, docetaxel, pulvomycin

## Abstract

Although docetaxel-based regimens are common and effective for early-stage triple-negative breast cancer (TNBC) treatment, acquired drug resistance frequently occurs. Therefore, a novel therapeutic strategy for docetaxel-resistant TNBC is urgently required. Signal transducer and activator of transcription 3 (STAT3) plays a pivotal role in the tumorigenesis and metastasis of numerous cancers, and STAT3 signaling is aberrantly activated in TNBC cells. In this study, a docetaxel-resistant TNBC cell line (MDA-MB-231-DTR) was established, and mechanisms for the antitumor activity of pulvomycin, a novel STAT3 inhibitor isolated from marine-derived actinomycete, were investigated. Levels of activated STAT3 (p-STAT3 (Y705)) increased in docetaxel-resistant cells, and knockdown of STAT3 recovered the sensitivity to docetaxel in MDA-MB-231-DTR cells. Pulvomycin effectively inhibited the proliferation of both cell lines. In addition, pulvomycin suppressed the activation of STAT3 and subsequently induced G_0_/G_1_ cell cycle arrest and apoptosis. Pulvomycin also significantly inhibited the invasion and migration of MDA-MB-231-DTR cells through the modulation of epithelial-mesenchymal transition markers. In an MDA-MB-231-DTR-bearing xenograft mouse model, the combination of pulvomycin and docetaxel effectively inhibited tumor growth through STAT3 regulation. Thus, our findings demonstrate that the combination of docetaxel and STAT3 inhibitors is an effective strategy for overcoming docetaxel resistance in TNBC.

## 1. Introduction

Although cancer mortality rate has steadily decreased due to improvements in early diagnosis, the five-year survival rate for some types of cancers is unsatisfactory owing to a lack of specific therapeutic options [1,2]. Approximately 30% of all cancers in women are breast cancer (BC) and BC is the second leading cause of cancer-related mortality in women [3,4]. Resection surgery, radiation therapy, and conventional chemotherapies are the most common and effective therapeutic strategies for early-stage BC. Among newly diagnosed BCs, approximately 20% are referred to as triple-negative breast cancer (TNBC), which is absent of hormone receptors (estrogen receptor and progesterone receptor) and human epithelial growth factor receptor 2. TNBC is the most aggressive cancer type, with a high recurrence rate and high intractability because they do not respond to targeted therapies for hormone receptors [5,6]. Cytotoxic chemotherapies based on taxanes (e.g., paclitaxel and docetaxel) are a mainstay of TNBC treatment; docetaxel is more frequently used not only because of its higher activity but also its convenient dosing schedule [7,8]. However, taxanes are reported to cause severe side effects, such as temporary hair loss, vomiting, and peripheral neuropathy, due to their relatively low selectivity for cancer cells compared with normal cells [9]. Moreover, long-term treatment with taxanes is limited due to the emergence of acquired resistance within the treatment period [10,11]. Therefore, novel therapeutic strategies for patients with taxane resistance are urgently required.

Signal transducer and activator of transcription 3 (STAT3), a transcription factor and signaling molecule, regulates the transcription of genes involved in various cellular functions [12,13]. Mounting evidence suggests that STAT3 is overexpressed in TNBC cells and plays a pivotal role in their survival, progression, and metastasis [14,15]. Hence, the regulation of STAT3 signaling with STAT3 inhibitors might benefit patients with TNBC who do not respond to cytotoxic chemotherapies. Furthermore, recent studies have reported that (i) STAT3 inhibition can potentiate the antitumor activity of cisplatin [16]; (ii) inhibitor of Janus kinase 2 (JAK2) blocks STAT3 activation and, thus, overcome tamoxifen resistance [17]; and (iii) activation of STAT3 induces resistance to many targeted cancer therapies [18]. Therefore, we hypothesized that targeting STAT3 signaling is a promising approach for overcoming acquired-chemoresistance in TNBC. However, STAT3 inhibitors are not FDA-approved for TNBC due to lack of potency [19].

Pulvomycin was originally discovered as a macrolide antibiotic from *Streptomyces* sp. [20]. Even though it was reported that pulvomycin prevents bacterial translational elongation factor as a promising antimicrobial compound [21], its anticancer activity has been poorly studied. Previously, we isolated the macrocyclic lactone pulvomycin along with new congeners pulvomycins B-D from the marine-derived actinomycete *Streptomyces* sp. HRS33, which were collected from an estuary between the Han River and the Yellow Sea in the Republic of Korea [22]. Pulvomycin exhibited potent growth inhibitory activity against a panel of cancer cells with IC_50_ values of 0.8–4.1 µM [22]. However, molecular mechanisms involved in the antiproliferative activity of pulvomycin and whether it can affect resistant cancer cells have not been elucidated. Given the remarkable effect of pulvomycin on cancer cell growth, we have evaluated its activity on docetaxel-resistant TNBC cells.

Here, we present evidence supporting the role of STAT3 signaling in docetaxel-resistant TNBC cells. Moreover, we report a novel STAT3 inhibitor, pulvomycin, which exhibits promising antitumor activity against docetaxel-resistant TNBC cells both in in vitro cell culture and in vivo xenograft models. We further demonstrated that combining pulvomycin and docetaxel resulted in overcoming docetaxel resistance. These findings indicate that STAT3 can be used as a novel and promising target for treating docetaxel-resistant TNBC.

## 2. Materials and Methods

### 2.1. Kaplan–Meier Plotter Analysis

The Kaplan–Meier Plotter (http://kmplot.com/analysis/) is an online database of published microarray datasets that assesses the effect of 54 k genes (mRNA, miRNA, and protein) on survival in 21 cancer types. In this study, the Kaplan–Meier Plotter was used to evaluate the overall survival (OS) of patients with breast cancer. Hazard ratios (HRs) with 95% confidence intervals (CIs) and log rank *p*-values were also computed (access date: 21 December 2020).

### 2.2. Cell Culture and Chemicals

The human breast epithelial cell line (MCF10A) and human TNBC cell lines (Hs578T, MDA-MB-231, HCC38, and HCC1937) were provided by the American Type Culture Collection (Manassas, VA, USA). The docetaxel-resistant cell line MDA-MB-231-DTR was generated in vitro by culturing MDA-MB-231 cells with increasing doses (0.02–2 µM) of docetaxel. MCF10A cells were cultured in Dulbecco’s modified Eagle’s medium/Nutrient Mixture F-12 containing 5% donor horse serum, 100 ng/mL cholera toxin, 10 µg/mL human insulin, 20 ng/mL epidermal growth factor, 0.5 µg/mL hydrocortisone, and penicillin–streptomycin (sodium penicillin G: 100 units/mL; streptomycin: 100 µg/mL). TNBC cells were cultured in media, namely Dulbecco’s modified Eagle’s medium for MDA-MB-231 and Hs578T cells and Roswell Park Memorial Institute 1640 medium for HCC38 and HCC1937 cells, supplemented with penicillin–streptomycin and 10% fetal bovine serum at 37 °C in a humidified incubator with 5% carbon dioxide [23]. All reagents used for cell culture, including culture media, fetal bovine serum, trypsin-EDTA solution (1×), and penicillin–streptomycin solution (100×), were purchased from Gibco (Grand Island, NY, USA). Laemmli sample buffer (2×) and 2-mercaptoethanol were purchased from Bio-Rad Laboratories, Inc. (Hercules, CA, USA). Dimethyl sulfoxide (DMSO), bicinchoninic acid, copper (II) sulfate solution, bovine serum albumin (BSA), trichloroacetic acid, sulforhodamine B (SRB), and docetaxel were purchased from Sigma-Aldrich (St. Louis, MO, USA).

### 2.3. Western Blot Analysis

Total cell lysates were prepared in 2× sample loading buffer (250 mM Tris-HCl (pH 6.8), 10% glycerol, 4% sodium dodecyl sulfate (SDS), 2% β-mercaptoethanol, 0.006% bromophenol blue, 5 mM sodium orthovanadate, and 50 mM sodium fluoride; Bio-Rad, Hercules, CA, USA). Protein concentrations were quantified through the BCA method [24] using the BCA Protein Assay Kit (Thermo Fisher Scientific, Waltham, MA, USA). Equal amounts of protein (5–20 µg) were separated using 6–13% SDS-polyacrylamide gel electrophoresis (PAGE) and transferred to polyvinylidene fluoride membranes (Millipore, Bedford, MA, USA). The membranes were blocked with 5% BSA (Sigma-Aldrich) and probed with anti-phospho-STAT3 (Y705), anti-STAT3, anti-cyclin E, anti-CDK2, anti-cyclin D1, anti-CDK4, anti-CDK6, anti-survivin, anti-cleaved caspase-9, anti-cleaved caspase-3, anti-cleaved PARP (D214), and anti-vimentin antibodies (Cell Signaling Technology, Beverly, MA, USA); anti-β-actin horseradish peroxidase antibody (Santa Cruz Biotechnology, Dallas, TX, USA); anti-E-cadherin and anti-N-cadherin antibodies (BD Biosciences, San Jose, CA, USA); or anti-Ki-67 antibody (Abcam, Cambridge, UK). The blots were detected using the WEST-Queen detection system (iNtRON Biotechnology, Seongnam, Korea) [25].

### 2.4. SRB Assay (Cell Proliferation Assay)

Cell proliferation was evaluated using the SRB assay [26]. Briefly, cells were seeded in 96-well plates and incubated for 30 min (for zero-day controls) or treated with test compounds for 72 h. After incubation, cells were fixed, dried, and stained with 0.4% (*w*/*v*) SRB in 1% (*v*/*v*) acetic acid. The unbound dye was removed by washing, and stained cells were resuspended in 10 mM Tris (pH 10.0). Cell proliferation was determined by measuring the absorbance at 515 nm.

### 2.5. Plasmid Transfection

The TurboFectin Transfection Reagent (#TF81001; Origene, Rockville, MD, USA) was used to transfect parental MDA-MB-231 cells with pCMV6-STAT3 (#RC215836; Origene) or the pCMV6-Entry control vector (#PS100001; Origine). All transfection procedures were performed according to the manufacturer’s protocol. After 48 h, MDA-MB-231 cells were harvested for protein isolation.

### 2.6. MTT Assay (Cell Viability Assay)

Cell viability was evaluated using the MTT assay. Briefly, cells were seeded in 96-well plates. On the next day, cells were treated with the indicated concentrations of test compounds and incubated for 72 h. IC_50_ values were calculated through non-linear regression analysis using the TableCurve 2D v5.01 software (Systat Software Inc., San Jose, CA, USA). The combination effect was evaluated through combination index (CI) values, which were calculated as follows: CI = D_1/_(D_x_)_1_ + D_2/_(D_x_)_2_. D_1_ and D_2_ are the concentrations of the combined test compounds that achieve the expected effect. (D_x_)_1_ and (D_x_)_2_ are the concentrations that achieve similar effects when test compounds are used alone. CI values were compared with reference values according to Chou [27].

### 2.7. RNA Interference

RNA interference for STAT3 was performed using siRNA duplexes purchased from Bioneer Corporation (Daejeon, Korea). The coding strand for STAT3 was designed as follows: #1-sense CUC CAA CAU CUG UCA GAU G and antisense CAU CUG ACA GAU GUU GGA G; #2-sense UGU UCU CUG AGA CCC AUG A and antisense UCA UGG GUC UCA GAG AAC A; and #3-sense CUA UCU AAG CCC UAG GUU U and antisense AAA CCU AGG GCU UAG AUA G. MDA-MB-231-DTR cells were transfected with 10 nM siRNA duplexes using Lipofectamine RNAiMAX (Invitrogen, Grand Island, NY, USA) according to the manufacturer’s recommendations. Cells transfected with a control non-specific siRNA duplex were used as controls for direct comparison. After 48 h, MDA-MB-231-DTR cells were harvested for protein isolation.

### 2.8. Cell Cycle Analysis

Cell cycle dynamics were measured using flow cytometry. MDA-MB-231-DTR cells were treated with either vehicle (DMSO) or pulvomycin in a complete medium for the times indicated. After incubation, all adherent and floating cells were collected, washed twice with phosphate-buffered saline (PBS), and fixed in 70% cold ethanol overnight at −20 °C. The fixed cells were washed with cold PBS, resuspended in 100 µg/mL of RNase A in a shaker for 30 min, and stained with 50 µg/mL PI in the dark. Then, the fluorescence-activated cells were sorted, and their cellular DNA content was analyzed using a flow cytometer (FACSCalibur flow cytometer; BD Biosciences, San Jose, CA, USA). Data were calculated using the CellQuest v3.0.1 software (BD Biosciences), and distributions of cells in each phase of the cell cycle were displayed as histograms.

### 2.9. Annexin V-Fluorescein Isothiocyanate and PI Double Staining

MDA-MB-231-DTR cells were treated with pulvomycin for 48 h in complete medium and then stained with Annexin V-fluorescein isothiocyanate (V-FITC) and PI using the Annexin V-FITC apoptosis detection kit (BD Biosciences, San Jose, CA, USA) according to the manufacturer’s recommendations. Briefly, the incubated cells were harvested, washed twice in cold PBS, resuspended in 1× binding buffer, and treated with Annexin V-FITC and PI in the dark for 15 min. The stained cells were resuspended in 1× binding buffer and immediately analyzed using a flow cytometer (FACSCalibur flow cytometer; BD Biosciences).

### 2.10. Transwell Cell Invasion Assay

Twenty-four-well Transwell membrane inserts (diameter: 6.5 mm, pore size: 8 µm; Corning, Tewksbury, MA, USA) were coated with 10 µL of type I collagen (0.5 mg/mL, BD Biosciences, San Diego, CA, USA) and 20 µL of a 1:20 mixture of Matrigel (BD Biosciences) in PBS. After treatment with the indicated compounds for 24 h, MDA-MB-231 human TNBC cells (parent or docetaxel resistant) were harvested, resuspended in serum-free medium, and plated (2 × 10^5^ cells/chamber) in the upper chamber of the Matrigel-coated Transwell insert. Media containing 30% FBS were used as the chemoattractant in the lower chamber. After 24 h incubation, cells that had invaded outer surfaces of lower chambers were fixed and stained using the Diff-Quik Staining Kit (Sysmex, Kobe, Japan) and imaged using the Vectra v3.0 Automated Quantitative Pathology Imaging System (Perkin Elmer, Waltham, MA, USA). Representative images from three separate experiments were evaluated, and the number of invasive cells was semi-quantified using the ImageJ v1.52a software (National Institutes of Health, Bethesda, MD, USA) [28].

### 2.11. Wound Healing Assay (Cell Migration Assay)

MDA-MB-231 human TNBC cells (parent or docetaxel resistant) were grown to 90% confluence in a six-well plate. Subsequently, the cell monolayer was artificially wounded using the SPL Scar^TM^ Scratcher (SPL Life Sciences, Pocheon, Korea), and detached cells were removed after washing with PBS. Wounded cell cultures were then incubated with media containing 1% FBS and various concentrations of pulvomycin for 24 h. Wounds were photographed at 0 and 24 h under an inverted microscope (Olympus, Tokyo, Japan). Wound areas were quantified using the ImageJ v1.52a software and presented as percent cell migration (%) relative to the wound area at 0 h [29].

### 2.12. In Vivo Tumor Xenograft Model

All animal experiments were conducted according to the guidelines approved by the Seoul National University Institutional Animal Care and Use Committee (IACUC; permission number: SNU-200309-8, approval date: 25 March 2020). Female nude mice (BALB/c-nu, aged 4–5 weeks, weighing 18 g) were purchased from Central Laboratory Animal, Inc. (Seoul, Korea) and housed under pathogen-free conditions with a 12-h light–dark schedule. MDA-MB-231 or MDA-MB-231-DTR cells were injected subcutaneously into the flanks of mice (4 × 10^6^ cells in 200 µL PBS), and tumors were allowed to grow for 10 days until tumor volume reached approximately 100 mm^3^. The mice were randomized into vehicle control and treatment groups (*n* = 6) [30]. Vehicle control (normal saline with 0.5% (*w*/*v*) Tween 80), docetaxel (10 mg/kg body weight), pulvomycin (10 mg/kg body weight), or a combination of docetaxel (10 mg/kg body weight) and pulvomycin (10 mg/kg body weight) was administered intraperitoneally (i.p.) three times per week for 24 days; mice were euthanized 1 week later. Tumors were excised, weighed, and frozen for biochemical analysis. Tumor volume was measured using an electronic caliper according to the following formula: tumor volume (mm^3^) = 3.14 × length × width × height/6. Toxicity was evaluated based on body weight loss.

### 2.13. Ex Vivo Biochemical Analyses of Tumors

A portion of the frozen tumors excised from nude mice on the termination day of the experiment was homogenized using the BioMasher-II (Optima, Tokyo, Japan) in Complete Lysis Buffer (Active Motif, Carlsbad, CA, USA). Aliquots were stored at −80 °C. Protein expression in tumor lysates was quantified using the Bradford assay [31].

### 2.14. Statistical Analysis

Data are presented as the mean values ± standard deviation (SD) for the indicated number of independently performed experiments. Statistical significance (* *p* < 0.05, ** *p* < 0.01, and *** *p* < 0.001) was evaluated using Student’s *t*-test or one-way analysis of variance coupled with Dunnett’s *t*-test.

## 3. Results

### 3.1. The Effects of Pulvomycin on p-STAT3 Expression Levels and Proliferation of Human TNBC Cells

**Figure 1:**
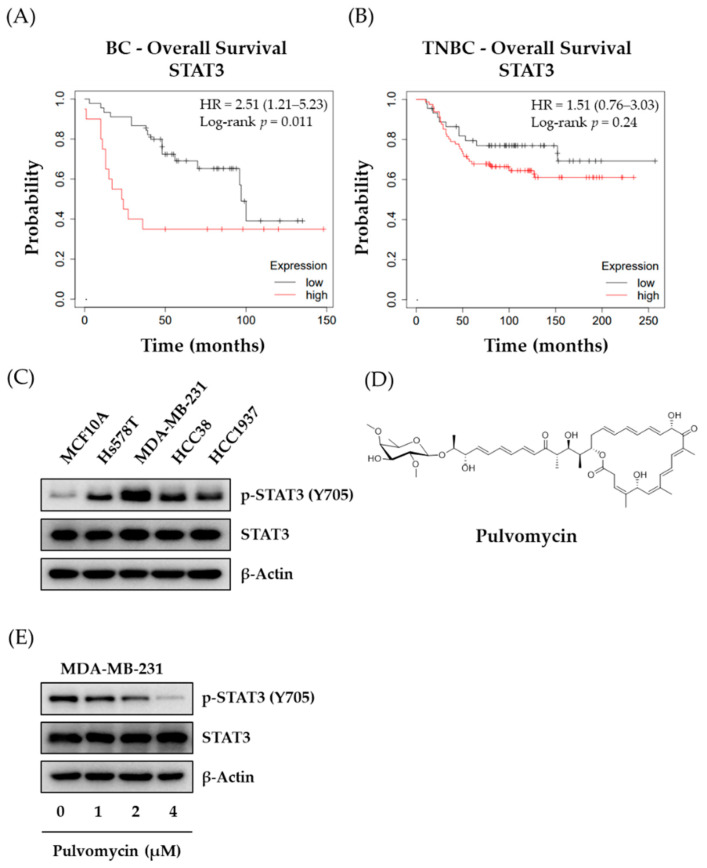
Relationship between signal transducer and activator of transcription 3 (STAT3) expression and overall survival (OS) in breast cancer, and the effect of pulvomycin on p-STAT3 in MDA-MB-231 cells. (**A**) The Kaplan–Meier survival curve for breast cancer-associated OS with STAT3 expression. (**B**) The Kaplan–Meier survival curve for TNBC-associated OS with STAT3 expression. (**C**) Levels of p-STAT3 expression in several TNBC cell lines were analyzed using Western blot analysis. β-Actin was used as an internal control. (**D**) Chemical structure of pulvomycin (**E**) MDA-MB-231 cells were treated with the indicated concentrations of pulvomycin for 24 h, and levels of p-STAT3 (Y705) and STAT3 expression were determined using Western blot analysis. β-Actin was used as an internal control.

To confirm the involvement STAT3 in TNBC, the clinical significance of STAT3 expression in patients with BC and TNBC was analyzed for overall survival (OS) using the Kaplan−Meier method. The auto-select best cutoff method was used to classify patients with breast cancer. As shown in Figure 1A,B, high levels of STAT3 expression were associated with a decreased probability for OS compared with low levels of STAT3 expression in both patients with BC and TNBC. These data indicate that in patients with TNBC, OS is inversely correlated with levels of STAT3 expression; thus, STAT3 might be a therapeutic target for TNBCs. Since STAT3 signaling is highly associated with TNBC cell proliferation [14], basal expression levels of activated-STAT3 (p-STAT3 [Y705]) were analyzed in a panel of TNBC cell lines (Figure 1C). All tested TNBC cell lines (Hs578T, MDA-MB-231, HCC38, and HCC1937) exhibited markedly higher p-STAT (Y705) expression when compared to the normal breast epithelial cell line (MCF10A). In particular, MDA-MB-231 cells exhibited the highest expression level of p-STAT3 (Y705); hence, the MDA-MB-231 cell line was selected as a representative cell line for TNBC in subsequent experiments for evaluating the effect of pulvomycin (Figure 1D). First, we assessed the effect of pulvomycin on the proliferation of TNBC cell lines. Pulvomycin exhibited potent antiproliferative activity against all tested TNBC cell lines (Table 1). In addition, the antiproliferative activity of pulvomycin against MCF10A was up to 20-fold lower than that against TNBC cells, which indicates that pulvomycin exhibits a relatively selective antiproliferative activity against TNBC cells compared with normal breast cells. STAT3 signaling is activated by the direct phosphorylation of a specific tyrosine residue (Y705) [32]. To confirm whether the antiproliferative activity of pulvomycin in TNBC cells is associated with STAT3 signaling, levels of p-STAT3 (Y705) expression in pulvomycin-treated MDA-MB-231 cells were evaluated through Western blot analysis. As shown in Figure 1E, the levels of p-STAT3 (Y705) were downregulated by pulvomycin in a concentration-dependent manner.

### 3.2. Establishment of Docetaxel-Resistant MDA-MB-231 Cells and Involement of STAT3 in Docetaxel Resistance

**Figure 2:**
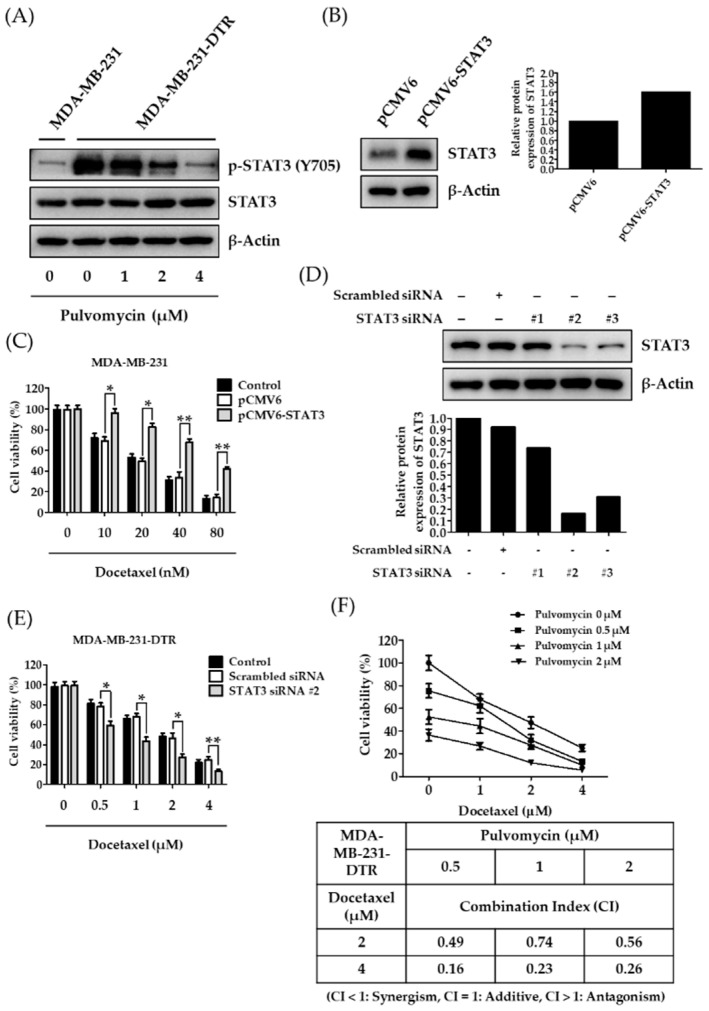
Signal transducer and activator of transcription 3 (STAT3)-dependent effects of docetaxel on the viability of human triple-negative breast cancer (TNBC) cells. (**A**) Cells were treated with the indicated concentrations of pulvomycin for 24 h, and levels of p-STAT3 (Y705) and STAT3 expression were determined using Western blot analysis. β-Actin was used as an internal control. (**B**) Western blot analysis of STAT3 expression in MDA-MB-231 cells 48 h after transfection with the control vector or STAT3 plasmid. β-Actin was used as an internal control. Relative intensities of indicated proteins were semi-quantitatively analyzed using NIH’s ImageJ v1.52a software. (**C**) The effect of docetaxel on the viability of MDA-MB-231 cells with the overexpression of STAT3. Cells transfected with either control vector or STAT3 plasmid were treated with various concentrations of docetaxel for 72 h, and cell viability was measured using the MTT assay. Data are presented as the mean ± SD (*n* = 3). * *p* < 0.05 and ** *p* < 0.01 compared with the vector-transfected control. (**D**) MDA-MB-231-DTR cells were transfected with scrambled siRNA or STAT3 siRNA for 48 h, and levels of STAT3 expression were determined using Western blot analysis. β-Actin was used as an internal control. Relative intensities of indicated proteins were semi-quantitatively analyzed using NIH’s ImageJ v1.52a software. (**E**) The effect of docetaxel on the viability of MDA-MB-231-DTR cells with the knockdown of STAT3. Cells transfected with either scrambled siRNA or STAT3 siRNA #2 were treated with various concentrations of docetaxel for 72 h, and cell viability was measured using the MTT assay. Data are presented as the mean ± SD (*n* = 3). * *p* < 0.05 and ** *p* < 0.01 compared with the vector-transfected control. (**F**) Cell viability was measured after combined pulvomycin and docetaxel treatment for 72 h in MDA-MB-231-DTR cells. Based on cell viability results, CI values were calculated to demonstrate the effect of the drug combination on MDA-MB-231-DTR cells.

Clinical evidence suggests that acquired resistance to previously treated chemotherapeutics is the major cause of treatment failure [33,34]. Although docetaxel elicits favorable initial responses against patients with TNBC, acquired resistance to docetaxel occurs after one year of treatment [35]. To further elucidate mechanisms of docetaxel resistance in TNBC cells, docetaxel-resistant MDA-MB-231 cells (MDA-MB-231-DTR) were established through continuous exposure of drug-sensitive MDA-MB-231 cells to docetaxel. MDA-MB-231-DTR cells exhibited approximately 90-fold resistance to docetaxel compared to parental MDA-MB-231 cells (Table 2). However, pulvomycin exerted potent antiproliferative activity with similar IC_50_ values in both cell lines. These data suggest that pulvomycin can overcome docetaxel resistance in MDA-MB-231-DTR cells. STAT3 signaling was reported to mediate drug resistance in various types of cancers [16,17,18]. Therefore, we evaluated levels of p-STAT3 (Y705) expression to confirm whether STAT3 signaling is involved in docetaxel resistance. MDA-MB-231-DTR cells exhibited p-STAT3 (Y705) overexpression compared to their parent cells; p-STAT3 (Y705) overexpression was effectively reverted by treatment with pulvomycin for 24 h (Figure 2A). To validate whether acquired resistance to docetaxel in human TNBCs is associated with STAT3 expression, docetaxel sensitive MDA-MB-231 cells were transfected with the STAT3 plasmid. The cytotoxic effect of docetaxel decreased in STAT3-induced cells compared to control vector-transfected cells (Figure 2B,C). By contrast, STAT3 knockdown in docetaxel-resistant MDA-MB-231 cells with STAT3 siRNA recovered their sensitivity against docetaxel (Figure 2D,E). These data support the hypothesis that STAT3 plays a critical role in the docetaxel resistance of TNBC cells. We further evaluated whether pulvomycin could resensitize resistant cells to docetaxel. Treatment with docetaxel and pulvomycin exhibited higher antiproliferative activity compared to treatment with either drug alone, and the synergistic effects were calculated through CI analysis using the Chou–Talalay method [27] (Figure 2F).

### 3.3. Effects of Pulvomycin on Cell Cycle Regulation and Apoptosis in MDA-MB-231-DTR Cells

**Figure 3:**
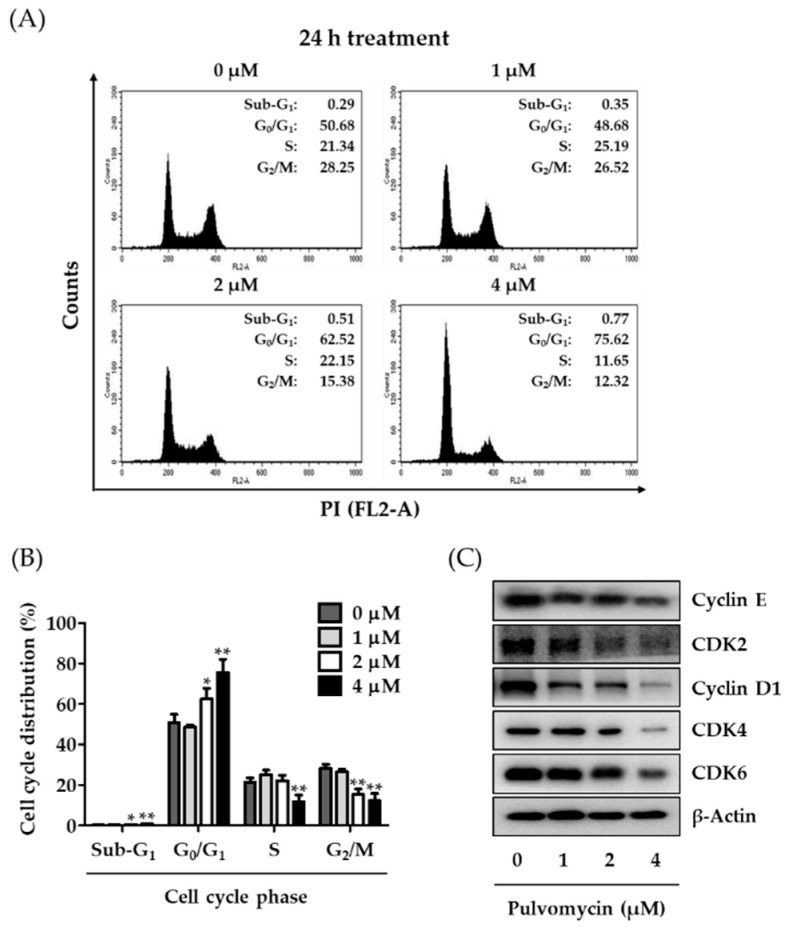
Effects of pulvomycin on cell cycle regulation. (**A**,**B**) MDA-MB-231-DTR cells were treated with the indicated concentrations of pulvomycin for 24 h and fixed with 70% ethanol for 24 h. Cell cycle distribution was determined in cells incubated with RNase A and PI for 30 min using flow cytometry. Data are presented as the mean ± SD (*n* = 3). * *p* < 0.05 and ** *p* < 0.01 compared with the control. (**C**) MDA-MB-231-DTR cells were treated with the indicated concentrations of pulvomycin for 24 h, and levels of cyclin E, CDK2, cyclin D1, CDK4, and CDK6 expression were determined using Western blot analysis. β-Actin was used as an internal control.

**Figure 4:**
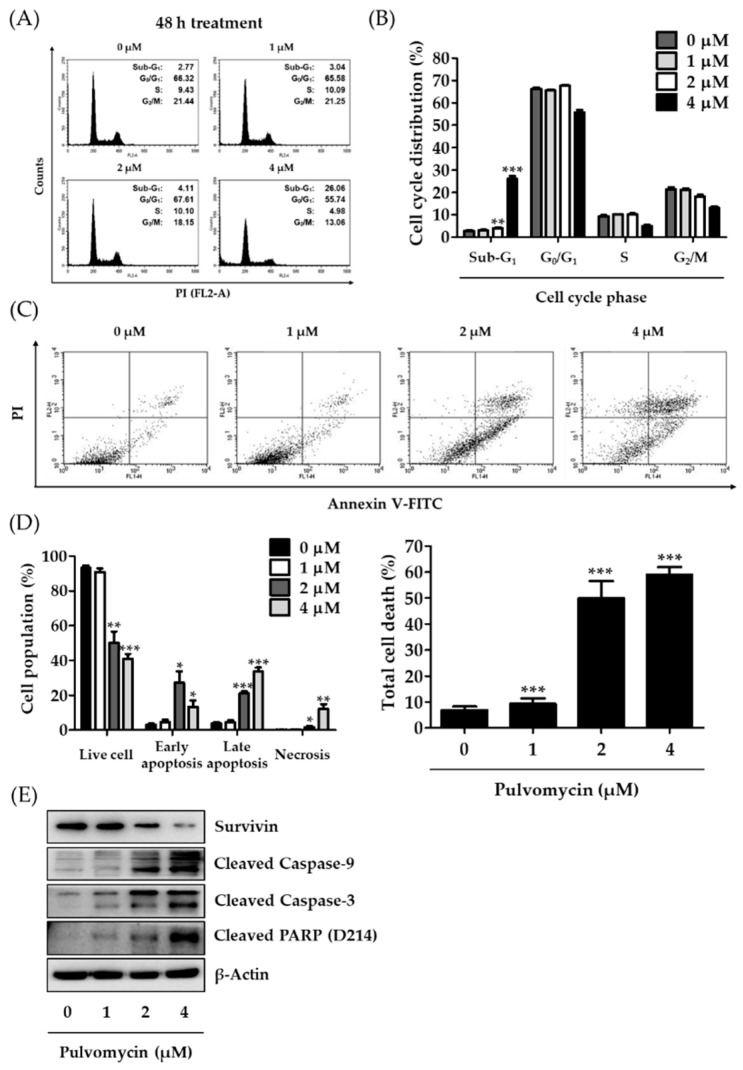
Effects of pulvomycin on apoptotic cell death. (**A**,**B**) MDA-MB-231-DTR cells were treated with the indicated concentrations of pulvomycin for 48 h and fixed with 70% ethanol for 24 h. After incubation with RNase A and PI for 30 min, cell cycle distribution was determined using flow cytometry. Data are presented as the mean ± SD (*n* = 3). ** *p* < 0.01 and *** *p* < 0.001 compared with the control. (**C**,**D**) MDA-MB-231-DTR cells were treated with the indicated concentrations of pulvomycin for 48 h and stained with Annexin V-fluorescein isothiocyanate (V-FITC) and PI. Annexin V/PI-positive cells were analyzed using flow cytometry to evaluate the apoptotic cell population. Data are presented as the mean ± SD (*n* = 3). * *p* < 0.05, ** *p* < 0.01, and *** *p* < 0.001 compared with the control. (**E**) MDA-MB-231-DTR cells were treated with the indicated concentrations of pulvomycin for 48 h, and levels of survivin, cleaved caspase-9, cleaved caspase-3, and cleaved poly (ADP-ribose) polymerase (PARP) (D214) expression were determined using Western blot analysis. β-Actin was used as an internal control.

The eukaryotic cell cycle is a series of events through which cells progress and divide. However, this universal process is aberrantly accelerated in most cancer cells [36,37]. In human TNBC cells, the activation of STAT3 signaling is closely related to cell cycle dysregulation; thus, inhibition of activated STAT3 might result in cell cycle arrest [38]. To confirm that the antiproliferative activity of pulvomycin is associated with STAT3-mediated cell cycle arrest, cell cycle phase distribution was determined using flow cytometry. Treatment with pulvomycin (1, 2, or 4 µM) for 24 h considerably increased the cell population in G_0_/G_1_ phase from 50.68% (control) to 75.62% (4 µM) (Figure 3A,B). The sequential activation of cyclin/CDK complexes regulates cell cycle progression [39,40]. To confirm whether the effect of pulvomycin on G_0_/G_1_ arrest is related to the regulation of cyclins and CDKs, levels of cyclins and CDKs involved in G_0_/G_1_ phase regulation were analyzed by Western blotting. As shown in Figure 3C, pulvomycin-treated MDA-MB-231-DTR cells exhibited a considerable decrease in the levels of all cyclins (cyclins E and D1) and CDKs (CDK2, CDK4, and CDK6). STAT3 signaling is also associated with apoptosis control in cancer cells [41,42]. Cells were treated with pulvomycin for 48 h, and cell cycle distribution was analyzed using flow cytometry. As depicted in Figure 4A,B, the population of cells in the sub-G_1_ phase (apoptotic cells) increased to 3.04%, 4.11%, and 26.06% after treatment with 1 µM, 2 µM, and 4 µM of pulvomycin for 48 h, respectively. These data suggest that prolonged exposure to pulvomycin may induce apoptosis in MDA-MB-231-DTR cells. To support this hypothesis, we performed flow cytometric analysis after double staining cells with Annexin V-FITC/PI. As shown in Figure 4C,D, populations of apoptotic cells, including those in early and late apoptosis, increased after treatment with pulvomycin for 48 h. To further confirm whether the induction of apoptosis by pulvomycin correlates with the regulation of apoptosis-related proteins, we performed Western blot analysis. Pulvomycin treatment for 48 h downregulated levels of survivin, a member of the inhibitors of apoptosis protein family, and upregulated levels of cleaved caspase-9, cleaved caspase-3, and cleaved poly (ADP-ribose) polymerase (PARP) (Figure 4E).

### 3.4. Effects of Pulvomycin on Cell Invasion, Migration, and Epithelial-Mesenchymal Transition in MDA-MB-231-DTR Cells

**Figure 5:**
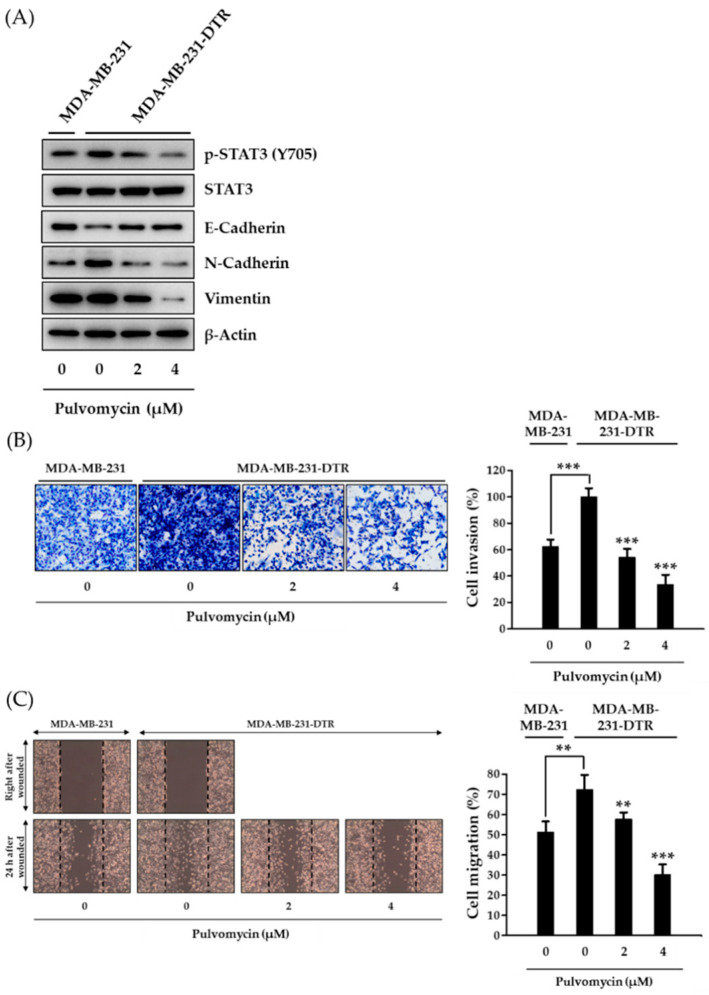
The effects of pulvomycin on epithelial-mesenchymal transition (EMT)-mediated cell invasion and migration. (**A**) The cells were treated with the indicated concentrations of pulvomycin for 24 h, and levels of p-STAT3 (Y705), STAT3, E-cadherin, N-cadherin, and vimentin expression were determined using Western blot analysis. β-Actin was used as an internal control. (**B**) The cells were pretreated with pulvomycin at the indicated concentration for 24 h, reseeded into the upper chambers of Transwell inserts, and incubated for 24 h. Cells that invaded the lower chambers were fixed, stained, imaged (left), and quantified using NIH’s ImageJ v1.52a software (right). Data are presented as the mean ± SD (*n* = 3). *** *p* < 0.001 compared with the control. (**C**) Cell monolayers were mechanically scratched and treated with pulvomycin for 24 h. Representative light microscopy images of wound closure are shown (left). Wound areas were quantified using NIH’s ImageJ v1.52a software (right). Data are presented as the mean ± SD (*n* = 3). ** *p* < 0.01 and *** *p* < 0.001 compared with the control.

Metastasis is responsible for almost 90% of cancer-related deaths, and BC is one of the most metastatic cancers, with metastasis rates reaching approximately 40% [43]. Moreover, metastatic potential increases when cancer cells acquire resistance [44]. Because STAT3 target genes are involved in critical steps of metastasis [45], the metastatic potential of MDA-MB-231-DTR cells was evaluated by analyzing epithelial-mesenchymal transition (EMT) biomarkers. In docetaxel-resistant MDA-MB-231 cells, E-cadherin (an epithelial marker for cell junctions) was downregulated, whereas N-cadherin (a mesenchymal marker) was upregulated. These aberrantly regulated EMT biomarkers were considerably downregulated by pulvomycin (Figure 5A). To further investigate the effects of pulvomycin on cancer cell metastasis, Transwell invasion and migration (wound healing) assays were performed. Consistently, MDA-MB-231-DTR cells exhibited increased invasion and migration, which were inhibited by treatment with pulvomycin for 24 h (Figure 5B,C). Taken together, these data indicate that pulvomycin exhibits anti-invasive and antimigration activities by regulating EMT biomarkers in docetaxel-resistant TNBC cells.

### 3.5. Antitumor Activity of Pulvomycin in an MDA-MB-231-DTR Cell-Implanted Xenograft Mouse Model

**Figure 6:**
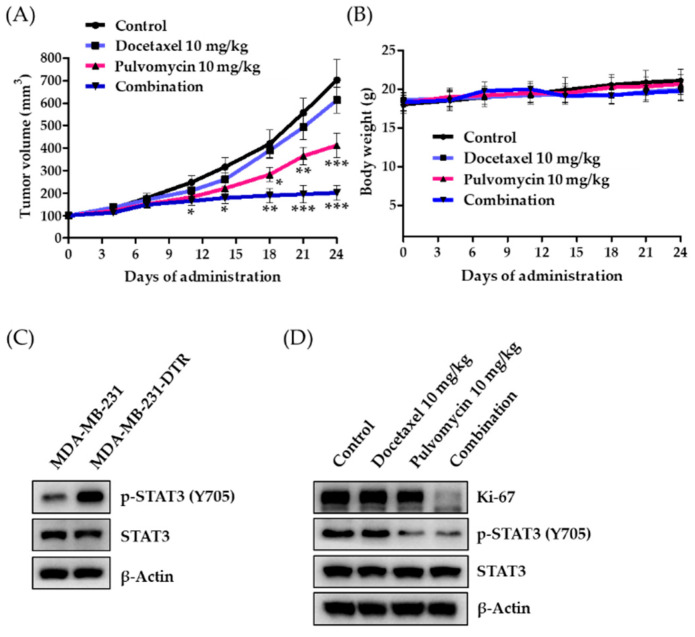
The combined administration of pulvomycin and docetaxel can overcome docetaxel resistance in an in vivo xenograft model. (**A**) Tumor volumes of the MDA-MB-231-DTR xenograft mouse model intraperitoneally administered vehicle, docetaxel, pulvomycin, or a combination of docetaxel (10 mg/kg body weight) and pulvomycin (10 mg/kg body weight) three times per week for 24 days were measured every 3–4 days with an electronic caliper. * *p* < 0.05, ** *p* < 0.01, and *** *p* < 0.001 were compared with the vehicle-administered control group. (**B**) Mouse body weights were measured every 3–4 days to assess general toxicity. (**C**) Small portions of tumor tissue from each group were homogenized in complete lysis buffer (Active Motif). Levels of p-STAT3 (Y705) and STAT3 expression were determined using Western blot analysis. β-Actin was used as an internal control. (**D**) Small portions of tumor tissue from each group were homogenized in complete lysis buffer (Active Motif). Levels of Ki-67, p-STAT3 (Y705), and STAT3 expression were determined using Western blot analysis. β-Actin was used as an internal control.

To further confirm the synergistic effect of pulvomycin and docetaxel on the growth of resistant tumors, antitumor activity was evaluated using a xenograft mouse model implanted with MDA-MB-231 or MDA-MB-231-DTR cells. When the tumor volume reached approximately 100 mm^3^, either vehicle (normal saline with 0.5% (*w*/*v*) Tween 80), docetaxel (10 mg/kg body weight), pulvomycin (10 mg/kg body weight), or a combination of docetaxel (10 mg/kg body weight) and pulvomycin (10 mg/kg body weight) were intraperitoneally administered three times a week for 24 days. Studies have shown that MDA-MB-231 cells respond well to docetaxel in a xenograft mouse model [46,47]. By contrast, our xenograft model, which was constructed using MDA-MB-231-DTR cells, exhibited negligible inhibition of tumor growth by docetaxel, whereas tumor growth was effectively suppressed by pulvomycin. Furthermore, the combination of pulvomycin and docetaxel exhibited remarkable antitumor efficacy in vivo (Figure 6A). No change in body weight and overt toxicity was observed in the drug-treated group compared to the vehicle-treated control group (Figure 6B). Consistent with our in vitro findings, additional biochemical analyses of MDA-MB-231 and MDA-MB-231-DTR tumor tissues from vehicle-treated groups revealed that activated STAT3 (p-STAT3 (Y705)) is considerably upregulated in resistant tumor tissues (Figure 6C). However, levels of p-STAT3 (Y705) and Ki-67, a cell proliferation marker, were downregulated in pulvomycin single- and combined- administration groups (Figure 6D). These results demonstrate that the combination of pulvomycin and docetaxel is a potential therapeutic strategy for patients with docetaxel-resistant TNBC.

## 4. Discussion

Despite improvements in early diagnosis techniques and the development of promising therapeutic strategies against cancer, cancer remains the major cause of death because of the acquired resistance to drugs and metastasis to other organs [48,49]. Notably, TNBC is the most recalcitrant type of cancer due to the absence of therapeutic targets. Docetaxel, a wide range cytotoxic chemotherapeutic agent, is the most effective strategy for both early- and late-stage TNBC. However, most TNBCs exhibit a high recurrence rate and increased resistance to docetaxel-based therapies [50]. Previously, we reported that STAT3 is overexpressed and aberrantly activated in human TNBC cells, and suggested that targeting STAT3 signaling using SLSI-1216, a potential STAT3 inhibitor, might be a compelling strategy for treating TNBCs [15]. In other reports, STAT3 signaling was found to be highly involved in the resistance of various cancer types [16,17,18]. However, the relationship between STAT3 and docetaxel resistance in TNBC is unclear. We hypothesize that the acquired resistance to docetaxel in TNBC might be overcome by targeting STAT3.

In this study, we established a docetaxel-resistant human TNBC cell line, namely MDA-MB-231-DTR, and found that STAT3 was abnormally activated in this resistant-cell line compared to the docetaxel-sensitive TNBC cell line. As STAT3 inhibitors have not been clinically approved for cancer therapy due to their relatively low potency or nonselective toxicity or both [51], we used pulvomycin, a STAT3 inhibitor isolated from marine-derived actinomycetes, to treat this aggressive type of cancer. Pulvomycin exhibited relatively selective growth inhibitory activity against human TNBC cells, namely Hs578T, MBA-MB-231, HCC38, and HCC1937, compared to normal epithelial breast cells (MCF10A) (i.e., 14–20-fold higher sensitivity for TNBC cells). Furthermore, the growth of the MDA-MB-231-DTR was effectively inhibited by pulvomycin. Analysis of the molecular mechanism revealed that the antiproliferative activity of pulvomycin against MDA-MB-231-DTR cells was partially associated with the induction of G_0_/G_1_ cell cycle arrest and apoptotic cell death. In addition, the involvement of STAT3 in docetaxel resistance was demonstrated using induction and knockdown experiments in TNBC cells.

Interestingly, the metastatic potential (invasion and migration) of docetaxel-resistant cells increased, indicating that STAT3 might be involved in both the resistance and metastatic potential of human TNBC cells. The increased metastatic potential was also effectively suppressed by pulvomycin treatment through the regulation of STAT3-mediated EMT. Given the remarkable activity of pulvomycin, we were intrigued with the possibility of its mitigation effect on docetaxel-resistant TNBC cells. The combination of pulvomycin and docetaxel had enhanced antiproliferative and antitumor activities both in vitro and in vivo. The enhanced activities on cancer cell growth by pulvomycin were partly associated with the inhibition of STAT3 activation. These results indicate that the use of the STAT3 inhibitor in combination with docetaxel might be a worthy strategy for treating docetaxel-resistant patients with TNBC. However, the detailed mechanisms of how pulvomycin regulate STAT3 activity warrant further study.

In summary, activated-STAT3 signaling is a driver of acquired-resistance to docetaxel in TNBC cells. Furthermore, the macrocyclic lactone pulvomycin is a novel STAT3 inhibitor with potent antitumor and antimigration activities against TNBC cells. The molecular mechanism of action for the activities of pulvomycin in docetaxel-resistant TNBC cells involves STAT3-mediated cell cycle arrest, apoptosis induction, and EMT pathway regulation. Thus, targeting STAT3 signaling through pulvomycin may be a plausible therapeutic approach for treating docetaxel-resistant TNBC.

## Figures and Tables

**Table 1:** Antiproliferative activities of pulvomycin against human triple-negative breast cancer (TNBC) cell lines.

IC_50_ (μM) ^a^	MCF10A	Hs578T	MDA-MB-231	HCC38	HCC1937
Pulvomycin	18.62	1.11	0.92	1.32	1.03
Docetaxel ^b^	0.23	0.01	0.02	0.02	0.05

^a^ Results are expressed as the calculated half maximal inhibitory concentration (IC_50_) of test compounds (μM) treated for 72 h. ^b^ Docetaxel was used as a positive control.

**Table 2:** Drug resistance profiles of MDA-MB-231 cells with resistance to docetaxel.

IC_50_ (μM) ^a^	MDA-MB-231	MDA-MB-231-DTR	Fold Difference ^b^
Pulvomycin	1.01	1.08	1.07
Docetaxel	0.02	1.83	91.50

^a^ Results are expressed as the calculated half maximal inhibitory concentration (IC_50_) of test compounds (μM) treated for 72 h. ^b^ The fold difference was calculated as the ratio of IC_50_ values between docetaxel-resistant-MDA-MB-231 and parent MDA-MB-231 cells.

## Data Availability

The data presented in this study are available in this article.
